# Lactate dehydrogenase activity staining demonstrates time-dependent immune cell infiltration in human ex-vivo burn-injured skin

**DOI:** 10.1038/s41598-021-00644-5

**Published:** 2021-10-28

**Authors:** Joshua Cuddihy, Gongjie Wu, Laptin Ho, Hiromi Kudo, Andreas Dannhorn, Sundhiya Mandalia, Declan Collins, Justin Weir, Ashley Spencer, Marcela Vizcaychipi, Zoltan Takats, Istvan Nagy

**Affiliations:** 1 Department of Surgery and Cancer, Imperial College London, Sir Alexander Fleming Building, South Kensington Campus, Exhibition Road, London, SW7 2BU UK; 2 Department of Metabolism, Digestion and Reproduction, Imperial College London, London, SW7 2BU UK; 3 Magill Department of Anaesthetics, Critical Care and Pain Medicine, Chelsea and Westminster Hospital NHS Foundation Trust, 369 Fulham Road, London, SW10 9NH UK; 4 Department of Burns Surgery, Chelsea and Westminster Hospital NHS Foundation Trust, 369 Fulham Road, London, SW10 9NH UK; 5 Research and Development, Chelsea and Westminster Hospital NHS Foundation Trust, 369 Fulham Road, London, SW10 9NH UK; 6 Department of Dermatology, Imperial College Healthcare NHS Trust, Du Cane Road, London, W12 0HS UK; 7 Department of Cellular Pathology, North West London Pathology, Charing Cross Hospital, London, W8 8RF UK

**Keywords:** Analytical biochemistry, Imaging, Mass spectrometry, Skin diseases, Inflammation, Macrophages

## Abstract

Burn injuries constitute one of the most serious accidental injuries. Increased metabolic rate is a hallmark feature of burn injury. Visualising lactate dehydrogenase (LDH) activity has been previously used to identify metabolic activity differences, hence cell viability and burn depth in burn skin. LDH activity was visualised in injured and uninjured skin from 38 sub-acute burn patients. LDH activity aided the identification of spatially correlating immunocompetent cells in a sub-group of six patients. Desorption Electrospray Ionisation Mass Spectrometry Imaging (DESI MSI) was used to describe relative lactate and pyruvate abundance in burned and uninjured tissue. LDH activity was significantly increased in the middle and deep regions of burnt skin compared with superficial areas in burnt skin and uninjured tissue and positively correlated with post-burn time. Regions of increased LDH activity showed high pyruvate and low lactate abundance when examined with DESI-MSI. Areas of increased LDH activity exhibited cellular infiltration, including CD3 + and CD4 + T-lymphocytes and CD68 + macrophages. Our data demonstrate a steady increase in functional LDH activity in sub-acute burn wounds linked to cellular infiltration. The cell types associated are related to tissue restructuring and inflammation. This region in burn wounds is likely the focus of dysregulated inflammation and hypermetabolism.

## Introduction

Burn injuries constitute one of the main sources of accidental tissue injury. They frequently result in significant adverse effects on survivors, such as poor wound healing, scarring, pain, susceptibility to infection, and long-term systemic complications^1^. Inflammatory responses alongside increased energy demand are hallmark features of burn injury and contribute to local and systemic adverse effects^1^. Abnormal inflammatory cell activity has been demonstrated in burn wounds and is linked to burn wound progression, systemic inflammatory responses, impaired vascularisation, delayed healing and infection^2^.

While inflammation significantly contributes to the increased energy demand, the biological processes and cellular and molecular mechanisms responsible for that inflammation-associated demand have not been fully established. Furthermore, regional changes in metabolic activity within burned skin are incompletely described though they likely contribute significantly to the inflammatory processes and clinical complications.

Assessing aerobic lactate dehydrogenase (LDH) activity in burn-injured tissues has been used to identify the depth of injury and highlight viable cells within the tissue^3–6^. This approach is based on aerobic LDH-mediated lactate-pyruvate conversion and concomitant conversion, by protons, of nitroblue tetrazolium to formazan that is a blue precipitate^3,6,7^. While the reaction shows oxygenated tissue compartments with cells exhibiting high energy production, the type of cells exhibiting formazan deposits in burn tissues have been incompletely identified so far. Thus, identifying the cell types which house LDH-mediated lactate-pyruvate conversion would significantly improve our currently incomplete understanding of the pathomechanisms of burn injury.

In this paper we use LDH activity staining to describe the differences between burn and non-burn skin in patients requiring burn wound debridement in the sub-acute period following a thermal injury. We further describe how these differences change over time since injury, and the differences between mechanism of thermal injury. Finally, we correlate the spatial distribution of LDH activity with tissue lactate and pyruvate as identified by imaging mass spectrometry and immune cell infiltration to the skin. This study aims to enhance understanding of the inflammatory process in sub-acute burn wounds.

## Results

### A heterogeneous cohort of patients was recruited, reflecting the variety of patients typically presenting for burns care

Thirty-eight patients were recruited from a regional burns unit to the study, having sustained clinically assessed burns of mixed depth requiring surgical intervention as part of usual clinical care. A range of patients was recruited (demographics presented in Table 1 and summarised in Table 2). The most common injury type was scalding (21/38). Male patients made up the majority of participants (24/38). There was a broad range of patient age (IQR 27–60 years) and time since injury (IQR 4–11 days). Total body surface area (TBSA) percentage affected was relatively low (median 2.25%, IQR 1–3.65%). During surgical intervention, tissue biopsies were taken from the burn and control wound. An example of tissue sampling is provided in supporting information 1 Figure 1.

**Table 1:** Demographics of recruited patients.

Age	Gender	Type of Burn	% TBSA	Age of Burn (days)	Site of burn biopsy	Site of control biopsy
41	M	Scald	2	1	Right dorsum foot	Right thigh
31	M	Scald	1	2	Right forearm*	Right thigh
33	M	Scald	2.5	2	Right supraclavicular fossa	Right thigh
58	M	Flame	14	2	Anterior abdomen**	Left groin**
26	M	Contact	0.25	3	Left knee	Left thigh
25	F	Flame	3	3	Anterior chest wall	Left flank
87	M	Scald	3	3	Left lateral forearm**	Left groin**
33	M	Scald	2.5	4	left forearm	Left thigh
19	F	Scald	1	4	Right dorsum foot	Right thigh
42	M	Scald	2	4	Left dorsum foot	Left thigh
23	F	Scald	1	4	Left anterior forearm	Left thigh
85	F	Contact	7	4	Right anterior shin	Right thigh
58	F	Contact	2	5	Left forearm	Left thigh
67	M	Scald	1	5	Right dorsum foot	Right thigh
23	F	Contact	1	5	Left thigh	Left groin
31	M	Contact	1.5	5	Left lateral lower leg*	Left thigh
58	F	Scald	3.5	5	Right anterior thigh	Right thigh
23	M	Contact	1	7	Left forearm	Left thigh
55	M	Scald	3	7	Right lateral lower leg*	Right thigh
58	F	Scald	6	7	Left lateral abdomen**	Left thigh**
53	M	Scald	1	7	Left Dorsum foot**	Left groin**
71	M	Scald	3	8	Right posterior thigh*	Right thigh
60	M	Flame	15	9	Left lateral leg below knee	Left thigh
61	F	Scald	3	9	Left forearm	Left thigh
28	M	Flame	1	9	Right side abdomen	Right thigh
31	M	Scald	8.5	9	Right anterior thigh	Left thigh
50	F	Scald	1	10	Right thigh	Right thigh
55	M	Flame	7	11	Left shin	Left thigh
63	M	Contact	4	11	Left medial proximal forearm	Left thigh
26	M	Scald	1	13	Lower back	Right thigh
41	F	Flame	3	13	Right anterior arm**	Right thigh**
45	F	Contact	1.5	14	Left forearm	Right thigh
24	F	Scald	1	15	Right dorsum foot	Right thigh
76	M	Scald	2.5	15	Left anterior distal thigh	Left thigh
84	M	Flame	7	17	Right lateral shoulder	Right thigh
37	M	Scald	1	17	Right dorsum foot**	Right thigh**
70	M	Flame	4	19	Right buttock*	Right thigh
24	F	Flame	0.25	26	Right dorsum foot	Right buttock

Demographics of patients recruited to study, and biopsy site (burn and uninjured control). *Note* *indicates those patients and biopsy sites underdoing additional formalin fixing and paraffin embedding (FFPE) and H&E staining; **indicates those biopsy sites undergoing FFPE and immunohistochemistry staining.

**Table 2:** Summary of recruited patients.

Gender	Male	Female	
	24	14	

Mechanism of injury (n)	Flame	Scald	Contact
	9	21	8

	Range	IQR	Median
Age of patient (years)	23–87	27–60	46.7
Time since burn (days)	1–26	4–11.5	7
TBSA (%)	0.25–15	1–3.65	2.25

Summary of patient demographics. Note the commonest injury type was a scald injury, more males than females were recruited with a broad range of patient age, time since injury and relatively low total body surface area (TBSA) percentage. During surgical intervention, tissue biopsies were taken from burn and control wound.

The heterogeneity of patients recruited reflects the broad range of burn injury types and patients seen in clinical practice. Further, the differing times since injury at the time of recruitment and sampling reflects the varying healing times following burn injury. Hence, this cohort of patients represents complicated burn wounds that are not healing adequately and are therefore of particular interest.

Ideally, sampling at different time points for each patient would have given further representation of changes with varying stages of healing. This approach was not used in this study due to the pragmatic design comprising surgical intervention as per usual care with simultaneous recruitment and the potential complications of repeated biopsies. The range of patients recruited, and the differing nature of injuries partially addresses this issue.

### Aerobic LDH activity is increased in the middle and lower third of burnt skin

**Figure 1:**
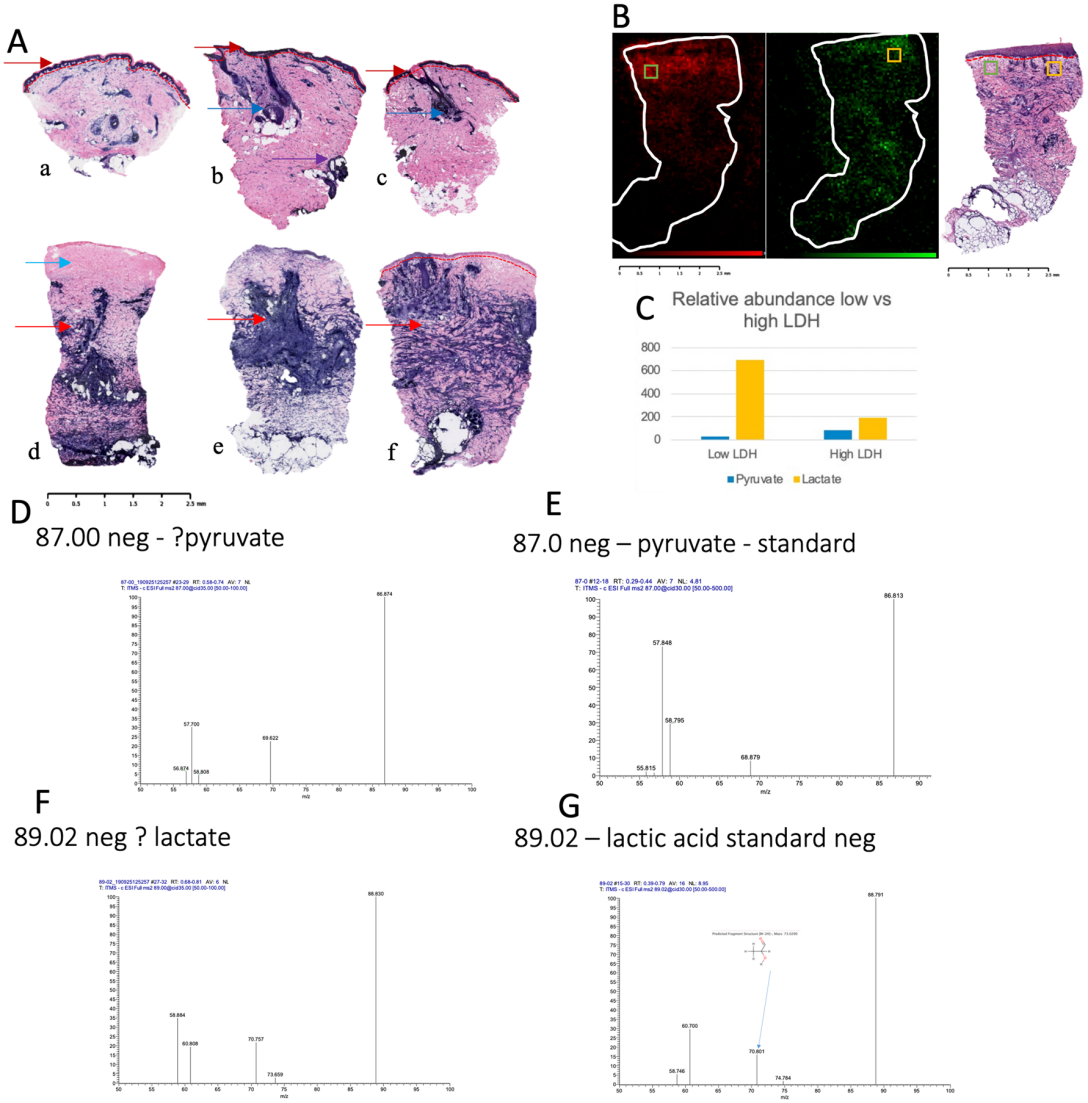
Examples of LDH stain pattern are shown in (**A**). All images at 50X magnification with 2.5 mm scale bar: (a–c) typical appearance of control skin with heavy LDH staining epidermis (dark red arrows), hair follicles (dark blue arrows), and sweat glands (purple arrow); (d–f—burn skin sections) (d) burn skin section with “classic” LDH staining—note complete absence of LDH in superficial part of tissue(light blue arrow); (e–g) burn skin sections showing non-linear LDH pattern of distribution in papillary and reticular dermis (red arrows). Red dotted line indicates epidermo-dermal junction when seen. Note epidermal cells not visualised in (e) or (g). (**B**) Left image (red): Desorption Electrospray Ionisation mass spectrometry image (DESI MSI) of m/z 89.0246 (lactic acid) for burn sample. Middle image (green): DESI MSI of m/z 87.0085 (pyruvate in same sample. Right image: LDH stain from same sample at 50 × magnification. Red dotted line indicates epidermo-dermal junction. Note the relative increase of lactate (in red) in the upper left region of the tissue corresponding with relatively less LDH stain update. White outline on mass spectrometry images indicates outline of tissue area. Scale bar shown. Note the spatial corelation between LDH staining and pyruvate and the anti-colocalization to lactate. Control skin shown in supplementary information. (**C**) Relative abundance for low LDH (green box In **B**) vs high LDH (yellow box in **B**) of lactic acid (m/z 89.026) and pyruvate (m/z 87.0085) in negative ion mode. (**D-G**) Fragmentation pattern of ? Pyruvate (m/z 87.00) (**D**) and ? Lactate (m/z 89.02) (**F**) compared against fragmentation pattern of commercially purchased pyruvate standard (m/z 87.0) (**E**) and lactic acid (m/z 89.02) (**G**) in negative ion mode. Note the similar fragmentation patterns, indicating identical molecular structures.

Visual inspection of LDH staining in control and burn sections revealed a difference between the distribution and degree of formazan deposits (Fig. 1A). Control skin samples had a typical appearance (Fig. 1A) in keeping with previous studies, principally with aerobic LDH expression in the epidermis and skin appendages, reflecting the high metabolism and turnover of these cell types^3,4^. In contrast, the formazan deposits, except in a few samples (3/38), were not present in the epidermis of the burnt skin. (all raw images presented in supporting information).

### LDH staining corresponds with low lactate and high pyruvate abundance in the burnt skin

To confirm that the formazan deposits are due to increased LDH activity, the distribution of formazan deposits was compared with DESI mass spectrometry images showing the distribution of lactate (m/z 89.0246 M-H^+^) and pyruvate (m/z 87.0085 M-H^+^) both in control and burn tissues. This comparison confirmed that formazan deposits were present in high pyruvate and low lactate areas in control and burn tissues. (Fig. 1B, C) Lactate and pyruvate presence in tissue was confirmed through tandem mass spectrometry of direct infusion electrospray ionisation tandem mass spectrometry. The fragmentation pattern of commercially available lactate and pyruvate was compared against tissue origin lactate and pyruvate, with the fragmentation patterns highly correlating. (Fig. 1D–G) This finding strongly supports the use of this LDH staining methodology to identify aerobic LDH activity in tissues. Further, this finding also indicates the value of DESI-MSI assessment of metabolite distribution in burn tissues to describe molecular pathological mechanisms.

### Pattern of LDH activity in burn and uninjured skin

In keeping with previously published work^3–5^, uninjured skin exhibited dense staining in the epidermis and around hair follicles and sweat glands. Burn samples exhibited a markedly different pattern, with lower levels of staining in the superficial regions of the tissue.

**Figure 2:**
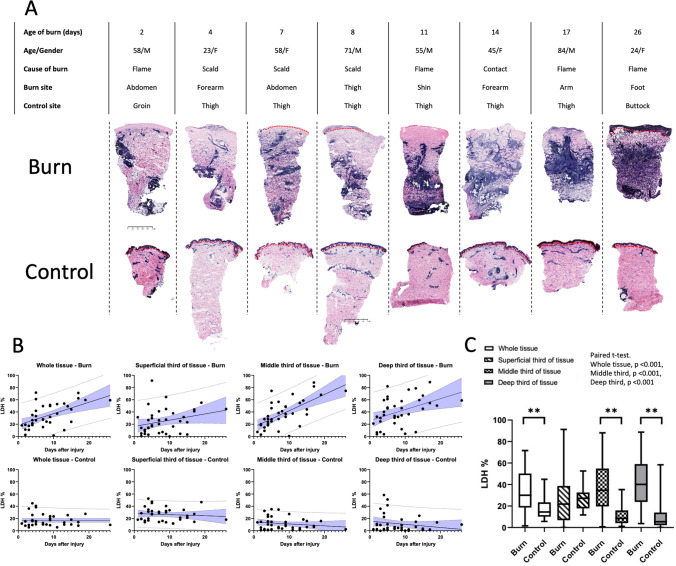
(**A**) Example images of changing LDH staining distribution of burns of older age compared against matched control skin. Demographics of patient, injury type, time since injury and body part section taaken from presented in table. Note the increasing extent of LDH staining (purple) in middle and lower depths of tissue in burn tissue with time since injury and non-linear pattern of this staining. In control skin, more typical pattern of staining is seen as described in Fig. 1(A). Red dotted line indicates epidermo-dermal junction. Note the absence of epidermal cells in 5/8 of the burn sections. (**B**) Time since injury and relationship with LDH stain percentage across whole tissue, superficial, middle and deep third burn vs. control skin is presented. Linear regression line of best fit is presented with 95% confidence interval, 95% prediction limits visualised. See supporting information 4, Table 2 for summary table showing slope, proportion of variance explained by the regression line (R^2^) together and p value, which accompanies Fig. 2B. (**C**) Box and whisker plot demonstrating average LDH percentage (median, IQR and range) for burn vs. control across whole tissue, superficial third, middle third and deep third of tissue is shown in (**D**) (n = 38, paired t test). **indicates p value of < 0.05.

Further, intense staining appeared in the reticular dermis and the papillary dermis in the majority of samples. (Typical staining patterns presented in Fig. 2A). Notably, a tendency of higher density of formazan deposits was observed in the samples of older age burns. The distribution pattern of the formazan deposits over time since injury suggests LDH activity might be associated with a cellular shift towards tissue remodelling from inflammation.

Image analysis confirmed that (except for the superficial third of the tissue) burn samples had significantly greater percentage areas that exhibited formazan deposits compared to control skin. Mean averages and p values calculated from paired t test: 34.08% vs. 17.05% (whole tissue: burn vs. control, p < 0.001), 24.8% vs. 26.63 (superficial third: burn vs. control, p = 0.55), 37.46% vs. 11.71% (middle third: burn vs. control, p < 0.001), 40.77% vs. 11.41% (deep third: burn vs. control, p < 0.001). See Table 3 for further detail. The heavily LDH stained healthy epidermis explains the non-significant difference in the superficial third of the skin in control skin compared with burnt skin. This finding supports the initial visual inspection. It suggests that LDH enzyme activity is significantly different between burn and control skin, particularly in the middle and deep third of the skin.

**Table 3:** Summary of regional LDH percentage analysis.

Sample	Burn	Control	Burn	Control	Burn	Control	Burn	Control
Tissue region	Whole	Whole	Superficial third	Superficial third	Middle third	Middle third	Deep third	Deep third
Mean LDH %	34.08	17.05	24.84	26.63	37.45	11.71	40.77	11.41
paired t test p value		** < 0.001**		0.55		** < 0.001**		** < 0.001**
SD	12.14	17.73	20.35	10.81	22.76	9.83	23.29	14.36
Median LDH %	30	14.42	21.76	27.19	34.65	8.57	40.13	5.365
IQR	18.7–49.7	10.1–22.5	7.5–36.9	18.1–32.3	20.1–53.2	4.2–15.8	25.9–58.6	2.2–13

N = 36. Each p value calculated using paired-t test with each burn sample paired with the control sample from the same patient. *LDH* lactate dehydrogenase activity staining, *SD* standard deviation, *IQR* interquartile range.

Bold numeric indicates p-value of < 0.05.

### Aerobic LDH activity exhibits a positive correlation with the age of the burn

The areas with formazan deposit in burn samples did correlate with the age of the injury when the entire section was assessed. The middle and deep thirds of the tissues exhibited a strong positive correlation with LDH expression and time since burn injury, reaching statistical significance, most markedly in the middle third (Fig. 2B, C, supporting information 4, Table 2).

### Aerobic LDH activity is not significantly different between burns of different aetiologies

LDH activity staining was increased in burns caused by flame compared with burn caused contact or scald across all regions of tissue in burn samples (supporting information 4, table 3). When analysed using the Kruskal wallis test, this did not reach statistical significance (p value > 0.05).

### Inflammatory cells are abundant in burn skin and regions of high aerobic LDH expression

To help identify cells associated with the increased LDH activity in burn samples, formalin-fixed sections from 5 patients were processed for Haematoxylin–Eosin (H&E) staining. (Demographics presented in Table 4). The staining showed dilated and thrombosed blood vessels and oedematous papillary dermis at varying depths from the surface of the burn tissue for each patient. This staining also revealed cellular enrichments composed of neutrophils, lymphocytes and macrophages predominantly in the perivascular regions of the dermis, occasionally around hair follicles and eccrine glands in the burn tissues.

**Table 4:** Demographics of histological imaging patients undergoing haematoxylin and eosin (H&E) stain.

Age	Gender	Type of burn	% TBSA	Age of burn (days)	Site of burn biopsy	Site of control biopsy	Additional stains
31	M	Scald	1	2	Right forearm	Right thigh	Haematoxylin and eosin
31	M	Contact	1.5	5	Left lateral lower leg	Left thigh	Haematoxylin and eosin
55	M	Scald	3	7	Right lateral lower leg	Right thigh	Haematoxylin and eosin
71	M	Scald	3	8	Right posterior thigh	Right thigh	Haematoxylin and eosin
70	M	Flame	4	19	Right buttock	Right thigh	Haematoxylin and eosin

The samples taken from these patients were immediately fixed in formalin before being embedded in paraffin.

**Figure 3:**
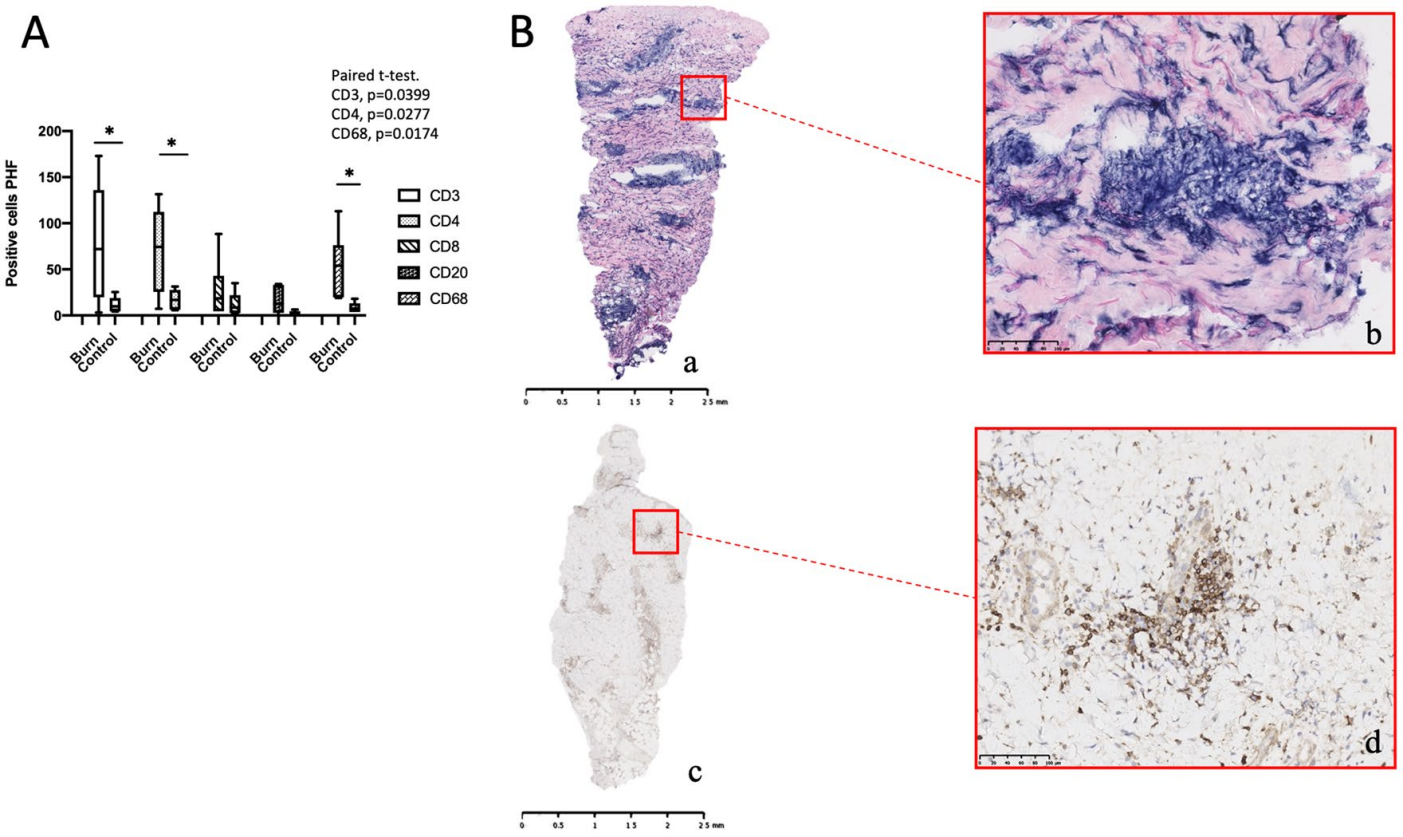
(**A**) Box and whisker plot indicating average number of cells per high powered field (PHF) (40X magnification) between burn and control skin. Cluster of differentiation (CD) 3, 4, 8, 20 and 68 stains. (n = 6). *denotes p value < 0.05 using paired t test. Note a tendency to increased cell density in all CD staining in burn tissue, although not reaching statistical significance in CD8 or CD20 staining. (**B**) Paired LDH stain (a) from 10 μm fresh frozen section and CD4^+^ stain (b) and 4 μm formalin fixed paraffin embedded section from adjacent area in same wound. Presented with scale bars. Note bands showing increased formazan deposits in reticular dermis of skin in regions anatomically correlating to regions increased CD4^+^ positive staining. Red boxes showing highlighted regions to demonstrate correlation between LDH staining and CD4^+^ staining. Scale bars shown in box.

To determine the cell types responsible for the enrichments next, anti-CD3, CD4, CD68, CD8 and CD20 antibodies were used for immunostaining on sections fixed in formalin from the adjacent area in burn and control skin from six patients. Demographic information relating to these patients are provided in Table 5. Visual inspection suggested enrichment in the burn samples for all cell lineages. Cell count confirmed a significant increase in CD3^+^ and CD4^+^ lymphocytes and CD68^+^ macrophages compared to non-burn tissue. (Fig. 3A) While the number of CD8^+^ and CD20^+^ expressing cells also increased, this increase did not reach statistical significance.

**Table 5:** Demographics of sub-group of patients undergoing IHC staining.

Age	Gender	Type of Burn	% TBSA	Age of Burn (days)	Site of burn biopsy	Site of control biopsy	Additional stains
58	M	Flame	14	2	Anterior abdomen	Left groin	IHC—CD3, 4, 8, 20, 68
87	M	Scald	3	3	Left lateral forearm	Left groin	IHC—CD3, 4, 8, 20, 68
58	F	Scald	6	7	Left lateral abdomen	Left thigh	IHC—CD3, 4, 8, 20, 68
53	M	Scald	1	7	Left Dorsum foot	Left groin	IHC—CD3, 4, 8, 20, 68
41	F	Flame	3	13	Right anterior arm	Right thigh	IHC—CD3, 4, 8, 20, 68
37	M	Scald	1	17	Right dorsum foot	Right thigh	IHC—CD3, 4, 8, 20, 68

The samples taken from these patients were immediately fixed in formalin before being embedded in paraffin.

### The distribution of CD3^+^ and CD4^+^ lymphocytes and CD68^+^ macrophages correlate to the areas of maximal aerobic LDH staining

The images of each cell lineage for each patient's burn and control sample were reviewed alongside the LDH stain from the same burn and control wounds. In all patients, anatomical features of the burnt skin were identified in the IHC stain and correlated with the same approximate anatomical region of the LDH stain (Fig. 3B). Regions of high LDH expression were consistent with regions of high cell density of CD3^+^, CD4^+^ and CD68^+^ markers, principally around dermal vasculature and eccrine glands in all burn tissues. Areas of lower LDH stain correlated with regions of lower cell density. In control skin, regions of high LDH around hair follicles and sweat glands within the skin's dermis were associated with areas of higher cell count. However, this was less apparent compared to the equivalent area in the burnt skin.

## Discussion

Cellular inflammation into burn wounds has been much studied and provides valuable insight into the differences between burn wounds and other skin pathologies and the link to the mechanisms behind burn wound complexity^8–11^. Many different cell types exist in a wound—the native cells preinjury, the infiltrating inflammatory cells (with or without invading pathogens) and the specialised cells that help restore structure and function^2,10^.

The importance of various immune cells in tissue injury healing is well recognised^8–10,12^ helping to coordinate the immune response with neutrophils, lymphocytes (T-cells), and macrophages described in thermal and non-thermal injury. Neutrophils are the first to arrive^13^. Their role of debridement of damaged tissue and protection against invading pathogens comes at the cost of further tissue loss through the utilisation of reactive oxidative species and proteases^14^. In this study, most samples were taken > 48 h after injury; therefore, the cell types seen in the skin in the sub-acute period were primarily investigated.

According to previous findings, at around 48 h post-injury, macrophages become the predominant cell^2,10,15–18^, helping to coordinate inflammasome responses to tissue damage to promote healing and limit tissue loss^18^. These cells help recognise damage associated molecular patterns (DAMPs) in the tissues, the consequences of non-infectious endogenous tissue breakdown^18^. In burn tissues, nucleotide-binding and oligomerization domain, leucine rich repeat and pyrin domain containing 3, (NLRP3) is the only inflammasome to recognise DAMPs^18,19^. Macrophage derived NLRP3 has been demonstrated in a murine knockout model by Vinaik et al. in 2020, to be essential in the recruitment of further macrophages to the burn wound, the polarisation of macrophages into pro-inflammatory states and release of pro-inflammatory mediators^18^. An increase in the number of macrophages was identified by the marker CD68 in areas of the cellular enrichment/formazan deposits in this study. Macrophages release cytokines and chemokines, stimulating granulation and new tissue growth, thus helping the healing wound progress from an inflammatory to a proliferative state^13^.

T-cells modulate the immune response of other cell types by releasing cytokines and are the most commonly identified lymphocyte subset in healing skin^12^. In addition to macrophages, a statistically significant increase in cells expressing the non-specific T lymphocyte marker CD3^+^ was detected. Like macrophages, T-cells play a vital role in the later stages of proliferation, early remodelling^13^ and help reduce the risk of wound infection, a common complication of burn wounds. Lymphocyte populations were observed to be more elevated in the dermis of burns patients who had sustained superficial dermal burns compared with deep dermal burns at 24 and 48 h post-burn^14^. This has been linked to the role of the superficial venous plexus being intact in the more superficial burn and the role this structure plays in lymphocyte delivery to the healing burn wound^14^.

T-helper cells have been identified as playing a pivotal role in burn wound healing and is reflected in our study with a significant increase in this cell type compared to non-burn skin and in areas of functional LDH activity. T-cells expressing Th1, Th2, and Th17 increased temporally following burn injury, with the most marked increase in Th17 (IL-17) compared with a non-burn injury^12^. Th17 cells, which release IL-17, play a pivotal part in chronic inflammation, auto-immunity and maintain the skin's barrier integrity^20^.

Bauhammer et al., in their 2019 paper describing an in vitro skin model, use LDH as a non-destructive marker of skin cell viability. In this work, the stability of LDH activity staining is investigated in varying conditions of the skin cell cultures^21^. The authors further describe the 5 main subunits of LDH enzyme existing in human tissue, comprising of different variants of the 2 tetramere subunits with LDH-5 being the most prevalent isotype in the epidermis of the skin, followed by LDH-4 and LDH-3. In the dermis of the skin, all 5 isotypes can be found with LDH-5 being the most prevalent. Whilst isotype LDH-3 is identified in the skin, it is also the most prevalent isotype found in white blood cells^21^. As described in the work of Gibson et al.^3^, in burn tissue in particular, the presence of LDH activity in migratory white blood cells as well as native skin cells in burn skin is consistent attempted tissue healing, and as such the non-specific measurement of these different LDH isotypes with staining method described does not limit the stains use in describing viability^3^.

In previous work, LDH staining has been used to identify burn depth with an absence of stain indicating loss of tissue and cellular viability^3–5^. Whilst pathologically this is of great interest, clinically, this is more challenging as wounds can be viable (have metabolically active tissue throughout all layers) while being full thickness injured and clinically problematic^4^. In the case of our cohort of patients, all had wounds requiring debridement and grafting based on clinical assessment. Yet, most had high levels of LDH staining, indicating the cellular "viability" of this tissue that clinically required debridement. This reflects that viability alone is not always a clinically meaningful outcome. For example, many wounds heal, but with significant scarring, delayed healing, wound infection or pain. Thus, LDH staining can further provide insight into differences in wounds beyond identifying viability alone in keeping with other published work^4^.

This study describes the staining distribution of functional LDH enzyme without measuring the enzyme presence in tissue directly. This is described as an advantage of the technique as it described presence of functional enzymes, not just the enzyme alone^3,5,7^. The demonstration of co-localised LDH enzyme with functional enzymatic staining in the same tissue would be an interesting additional measure to complement this study and could be considered for future work. However, the tissue region of most interest in this patient group is that of functional enzymatic activity as it denotes that region of tissue undergoing tissue healing processes and inflammation. It is this region that is at risk of wound progression and further tissue loss. Further, fresh frozen tissue is a difficult medium to perform direct enzymatic measurement but retains much of the biochemical information of significance detected by other modalities such as imaging mass spectrometry. For this reason, direct enzymatic staining was not chosen in this study.

Our study shows how LDH staining identifies the middle and deeper dermis as the regions of increased metabolic activity in sub-acute thermal skin burns. This knowledge directs other research modalities into this region and helps determine the factors contributing to tissue loss or restoration. In addition, our data confirm the distinct spatial separation of lactate and pyruvate in the regions consistent with LDH staining. These results show that LDH staining can be used to focus further mass spectrometry imaging and analysis on increasing our understanding of metabolic and cellular processes in healing burn tissue.

We demonstrated no significant difference in LDH activity in burn tissue of different aetiologies, although flame injury appeared to evoke the greatest response and scald injury, the least. This non-significance may be explained by the relatively small sample size when the 36 patients are separated into groups by aetiology, and the multiple confounding factors. However, the apparent increased LDH activity burns caused by flame injury may represent a difference in tissue response according to aetiology. A more pronounced initial thermal injury is likely caused by the more extreme temperatures evoked by flames compared to scald injury, and we postulate this may result in differences in the subsequent immune response. Imaging mass spectrometry analysis may prove insightful in describing differences in the chemical fingerprint of skin following thermal injury by different energy sources and aetiologies.

### Study limitations

Our study is limited through the multiple variables that exist within the clinical cohort of patients. Whilst this provides meaningful data representing real-world clinical challenges, it makes analytical techniques more difficult and introduces many confounding variables. The presence of paired control samples limits the impact of these confounding variables.

Burn tissue is difficult to section, making imaging analysis more challenging. The process of cryosectioning is time consuming, and requires experienced operators which may limit the expansion of this study into larger patient groups. Ideally depth from the superficial surface of the skin in micrometers would be used to describe differences in regional LDH activity. However, burn skin from patients, with varying degrees of oedema, inflammation, clotted blood and fat content did not consistently provide high quality sections allowing precise superficial to deep measurements. For this reason, a “thirds” approach was used which could reliably differentiate between superficial and deep regions. Further, obtaining near-identical sections for paired immunohistochemical staining and LDH activity staining was not achieved in this study due to the different preparation methods used. Whilst other groups have used different approaches allowing for formalin fixation and fresh frozen fixation from the same biopsy^5,6^, this was not possible in our study. Imaging mass spectrometry is best performed on minimally altered tissue, and immediate freezing is required to retain the biochemical fingerprint for analysis. Our approach of taking immediately adjacent biopsies that could undergoing formalin fixation prior to immunhistochemical analysis partially addressed this issue, as the samples were from the approximate same region of the burn wound. Despite this, the histological images obtained did not directly match up between frozen sections and formalin fixed, paraffin embedded sections. We therefore relied upon expert dermatohistopathologists to help identify correlated regions of tissue.

In summary, the combined assessment of LDH activity with functional staining, with DESI-MS imaging and immunostaining on tissue sections control and burn skin from burn-injured patients develops our understanding of the highly complex pathology of burn injury. This study finds that LDH staining is significantly increased in the middle and deeper regions of burn, increasing with time since injury. This increase is directly linked to CD4^+^ helper T lymphocytes and CD68^+^ macrophages infiltration into the dermis. The role of those cells in proliferation and tissue remodelling suggests that increased LDH activity is associated with those processes in burned skin. The link between mass spectrometry imaging analysis and LDH expression is further demonstrated in this study. This combined approach shows promise for research progress in the future, focussing attention on the deeper regions of burn tissue. The use of high throughout imaging mass spectrometry analytical techniques in combination with functional enzymatic staining promises to provide significant advances in our understanding of the biochemical processes involved in disordered burn wound inflammation and may direct further work into the development of novel therapies.

## Conclusion

LDH staining identifies significantly increased metabolic activity in the middle and deep third of burn than control skin. This further increases with time since injury in sub-acute thermal burn injury. This increase in LDH activity is related to cellular infiltration, including CD3^+^, CD4^+^, CD8^+^ lymphocytes, CD20^+^ B-cells and CD68^+^ macrophages, likely associated with inflammatory infiltration. In addition, it is connected to changes in the distribution of lactate and pyruvate, as identified by mass spectrometry imaging. It demonstrates how this technique can investigate this region of interest further and potentially other tissue types.

## Methods

### Patient recruitment and sample collection

Thirty-eight patients presented for surgical excision of burn wounds at a regional burn centre and were recruited *(IRAS reference: 189,005, REC:16/LO/0203).* Three-millimetre punch biopsies using sterile punch biopsy of clinically assessed mid-dermal burn skin were taken before wound debridement. Punch biopsies were also taken from an uninjured site, typically donor skin for grafting, and were used as control (supporting information 1, figure 2). Samples were frozen in dry ice before being stored at -80 degree centigrade. Tissues were prevented from thawing, embedded in ice or hydrogel^22^ and cryosectioned at 10 μm thickness for analysis. Patient demographic data are presented in Table 1 and summarised in Table 2.

### LDH assay

A modified LDH assay based upon Gibson et al. protocol was used^3^. Sections were allowed to dry at room temperature for 1–2 h. Slides were marked with a hydrophobic pen to surround the tissue margins and retain subsequent stains. Slides were washed two times in phosphate-buffered saline (PBS) before being incubated for 3–4 h at 37 °C in freshly prepared LDH solution (pH 8.0) containing stock polypep solution (5% polypep, 2 mM Gly-Gly, 0.75% NaCl, 60 mM lactic acid, 1.75 mg/ml b-nicotinamide adenine dinucleotide (NAD) and 3 mg/ml nitroblue tetrazolium. (Polypep: Sigma, Cat No. P5115, Gly-Gly: Sigma, Cat No. G3915, L-( +)-Lactic acid: Alfa Aesar, Cat No. L13242, Beta-Nicotinamide adenine dinucleotide hydrate: Sigma-Aldrich, Cat No. 43410, Nitroblue Tetrazolium: MP Biomedicals, Cat No.193999). (See supporting information 2 for protocol). Each experimental staining was accompanied with negative control staining, with lactic acid removed and with NAD removed, respectively. (See supporting information 2, figure 2). Following incubation, slides were washed with 50-degree centigrade tap water for 2 min twice and washed with PBS for two minutes. They were counterstained with eosin for 4 min, washed with PBS for one second, dehydrated with acetone for 30 s, 1:1 acetone:xylene for 1 min and xylene for 1 min. Slides were cover-slipped and scanned using NanoZoomer 2.0HT (Hamamatsu, Japan).

### LDH stain image analysis

Digital images were reviewed in NDP.scan 3.2.12 (Hamamatsu, Japan), orientated so superficial aspects of tissue were uppermost and exported at 2.5 × magnification as jpeg images. Images were analysed using Image J. A standardised measure was used to generate set pixels per micrometre and applied to all images. A purpose-built macro (available in supporting information 6) which colour deconvoluted the images to generate LDH stain (blue) and background stain Eosin (pink) was created. These distinct colours were homogenised, and threshold adjusted using identical settings. The total area of LDH stain and background stain was calculated. Tissues were divided into superficial, middle and deep thirds from top to bottom of the tissue using annotation of the whole tissue. Homogenised eosin staining formed "background" tissue, and homogenised LDH stain was expressed as a percentage of this area for the whole section and each of the tissue thirds. The percentage LDH stain compared to background was recorded for each section and each third for every patient burn and control sample. (Example in supporting information 3, figure 3 and table 1) The differences between burn and control, cause of burn and age since burn injury were measured.

### Histological imaging

5 out of 38 patients underwent an additional 3 mm biopsy of clinically assessed dermal burn (in addition to the biopsies described above). These samples were immediately fixed in formalin and stored at 4 °C. (Demographics presented in Table 2A). These samples were embedded in wax paraffin and sectioned to 3 μm thickness. Sections were analysed using haematoxylin and eosin (H&E). Slides were cover-slipped and scanned using NanoZoomer 2.0HT (Hamamatsu, Japan). Qualified dermatohistopathologists reviewed the images in NDP.scan 3.2.12 (Hamamatsu, Japan) and commented on the tissue morphology and inflammatory cells seen in the skin.

### Immunohistochemical staining

6 out of 38 patients underwent an additional 3 mm biopsy of clinically assessed dermal burn and 3 mm biopsy from control non-burn skin. These samples were immediately fixed in formalin and stored at 4°. (Demographics presented in Table 2B). Note these samples were immediately adjacent to the samples taken from the same patients that underwent LDH staining (supporting information 1, Figure 1). These samples were embedded in paraffin, and 3 μm thickness paraffin sections were pre-treated using heat mediated antigen retrieval. The sections were then incubated with CD3, CD4, CD8, CD20 and CD68 antibodies at room temperature and detected using an HRP conjugated compact polymer system. (CD3: Leica, Cat No. NCL-L-CD3-565, CD4: Leica, Cat No. PA0427, CD8: Leica, Cat No. PA0183, CD20: Dako, Cat No. M0755, CD68: Dako, Cat No. M087601). 3,3′-Diaminobenzidine (DAB) was used as the chromogen. The sections were then counterstained with haematoxylin and mounted with dibutyl phthalate in xylene (DPX). Immunohistochemical staining was performed on Leica BOND 3 autostaining system. Slides were cover-slipped and scanned using NanoZoomer 2.0HT (Hamamatsu, Japan). Manual cell counting for each CD marker was performed of cell dense tissue regions in 40 × magnification high-powered fields (HPF). Each HPF was measured from the superficial aspect of the tissue where tissue quality allowed. This was performed for all stains and in burn and paired control. Qualified dermatohistopathologists reviewed images, and tissue regions in the IHC were correlated with corresponding LDH stain regions.

### Statistics

For the percentage of LDH differences between burn skin and control skin, a paired t test was performed, and the mean differences are presented in the results. Causes of burn, namely contact, flame and scald, in relation to the percentage of LDH were non-parametric, partly due to the small sample size, and thus Kruskal Wallis test was performed. The relationship of age of burn with LDH utilises a linear regression model, with R2 value, 95% prediction limits and confidence intervals, and the proportion of variance shown. Cell count was performed manually, and due to the parametric nature, a paired t test was performed. Statistical significance is defined as p < 0.05. Data were analysed using Microsoft Excel (Microsoft, USA) and Prism 8 (GraphPad, USA).

### Mass spectrometry imaging and tandem mass spectrometry

Desorption Electrospray Ionisation Mass spectrometry imaging (DESI MSI) was performed to elucidate the spatial distributions of lactic and pyruvic acid in the tissues. Direct infusion electrospray tandem mass spectrometry was performed to validate the metabolites annotations made in the MSI dataset.

### Desorption electrospray ionisation (DESI) mass spectrometry imaging (MSI) methodology

DESI-MSI was performed on burned and non-burned skin sections from the patients in negative ion mode using Waters Xevo X2 QTOF instrument (Waters, Manchester, UK). The analysis was performed in the m/z range between 50 and 1500 and used a line-by-line sampling method using pre-set x and y coordinates. A spatial resolution of 50 μm by 50 μm was used for all experiments. To correct for mass shifts, MSI data was lockmass corrected to raffinose which was doped into the sprayer solvent, delivered with a flow rate of 1.5 μL/min and nebulised with nitrogen backpressure of 6 bar. The final concentration of raffinose in the sprayer solvent was ten ppm. Mass spectrometry images were processed and reviewed in HDImaging (waters).

### Tandem mass spectrometry

To validate metabolite annotations in the MSI data based on accurate mass measurements (m/z for lactic acid: 89.0246; pyruvate 87.0085), tissue sections were homogenised, extracted and the resulting solutions used for direct infusion (DI) tandem-MS experiments. Ten tissue sections of burned and control samples from 2 different patients were transferred into an extraction tube, and 1.5 mL 75% methanol and zirconia beads were added. The tissues were extracted using a tissue homogeniser (Precelllys 24, Bertin Technologies SAS, Montigny-le-Bretonneux, France). The homogenisation was performed in 2 cycles, each consisting of 45 s of shaking at 4600 revolutions per minute followed by 30 s of pause and another 45 s of shaking. Samples were centrifuged at 14,000 g for 20 min under refrigeration (PrismR, Labnet international Inc., Edison, NJ, USA) and the clear supernatants directly used for analysis. The resulting product ion spectra obtained from the tissue extracts were compared to pyruvate and lactate (Sigma-Aldrich S8636-100ML, L7022-5G). Tandem-MS experiments were performed on an LTQ XL instrument (Thermo Scientific, Bremen, Germany).

### Study approval

This clinical investigation was conducted according to the Declaration of Helsinki principles. The Health Research Authority-Westminster Research Ethics Committee, London UK, reviewed and approved the protocol. REC reference 16/LO/0203. Written informed consent was received from all participants before inclusion in the study. Participants are referred to by non-identifying characteristics throughout the study.

## Supplementary Information

Supplementary Information.

## Data Availability

The datasets generated during and/or analysed during the current study are available from the corresponding author on reasonable request.
