# Supplementary material for: Lactate dehydrogenase activity staining demonstrates time-dependent immune cell infiltration in human ex-vivo burn-injured skin
Source: Sci Rep. 2021 Oct 28;11:21249. doi: 10.1038/s41598-021-00644-5 (PMC8553775; doi:10.1038/s41598-021-00644-5)
Supplement: Supplementary file 1 — Supplementary Information. [file 41598_2021_644_MOESM1_ESM.docx]

**Supporting information**

**Supporting information 1: Figure 1.**

**
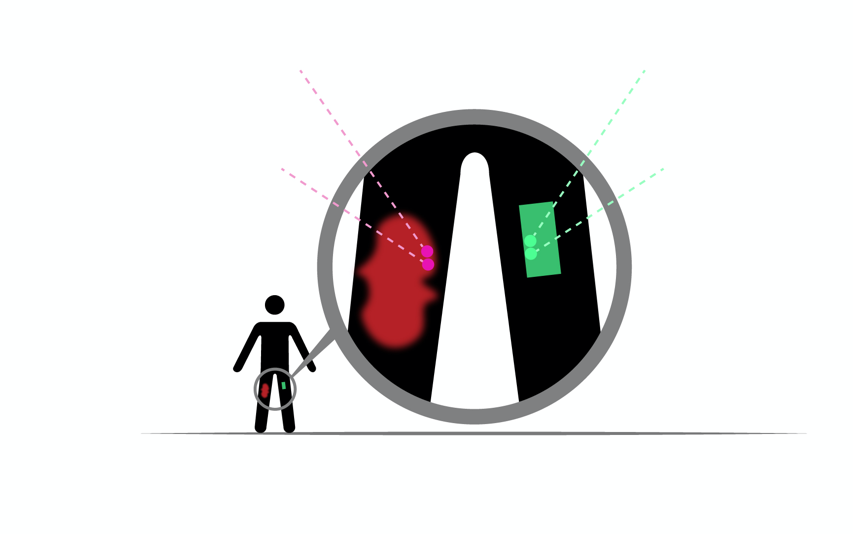
**

3mm punch biopsy burn (a)

3mm punch biopsy control (c)

3mm punch biopsy control (d)

3mm punch biopsy burn (b)

**Figure 1:** An example of intraoperative sampling approach. Recruited patients are sampled during planned burn wound debridement and grafting as part of usual care. Punch biopsies of 3mm diameter are taken from clinically assessed mid-dermal depth burn, and control sites (usually the donor site used for skin grafting) as indicated by a and c. These samples were immediately frozen in dry ice and stored at -80 degrees centigrade before processing. When further samples were taken for formalin fixation, these were simultaneously acquired from immediately adjacent areas in burn and control skin as indicated by b and d. Figure created using Adobe Inc. Adobe illustrator (2020), version 24.0.1. Available from <https://adobe.com/products/illustrator>. Created by Joshua Cuddihy with assistance from Mr Jacob Cuddihy.

**Supporting information 2: LDH Protocol:**

**Lactate Dehydrogenase (LDH) staining protocol**

Dry frozen sections at room temperature, 1hr

Marking with hydrophobic pen

Wash with PBS, 5min x2

Incubate with freshly prepared LDH solution, 3.5-4hrs, 37C, in the dark, use only supernatant

Stock polypep base solution

5% Polypep (5g/100ml)

2mM Gly-Gly(MW132.12) (26.4mg/100ml)

0.75% NaCl (0.75g/100ml)

Adjust to pH8.0, store at 4C with 2M NaOH (8g/100ml)

Add on the day

60mM lactic acid (MW 90.08) 54.048mg/10ml (4C)

1.75 mg/ml b-nicotinamide adenine dinucleotide NAD (light sensitive) 17.5mg/10ml (-20C)

Adjust to pH 8.0 with 2M NaOH (prep 9ml polypep solution + ca.300ul 2M NaOH)

NEG CON1 : Lactic Acid –

NEG CON2 : NAD –

Add immediately prior to use

30mg Nitroblue Tetrazolium /10ml polypep base solution (4C)

Wash with 50C tap water, 2min x2

Wash with PBS, 2min x 2 (move to NHS lab)

Counterstain with Eosin, 4min

Wash with PBS, 1sec

Dehydrate

Acetone, 30sec

Acetone:Xylene 1:1, 1min

Xylene, 1min

Coverslip

Supporting information 2: Figure 2

Positive and negative control sampling for LDH staining methodology


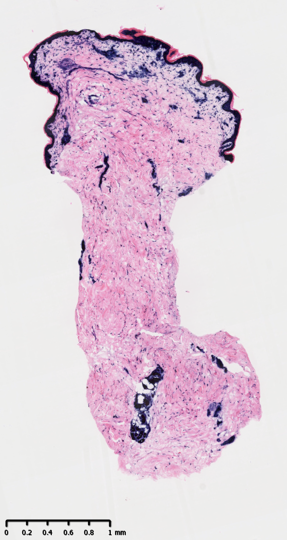

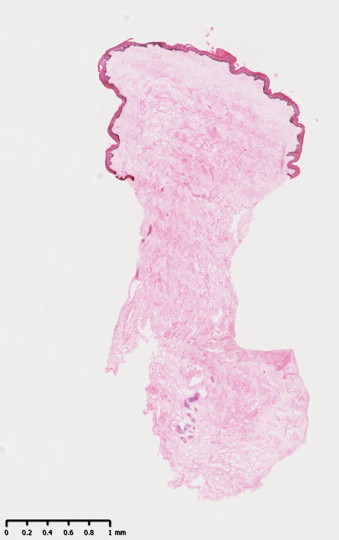

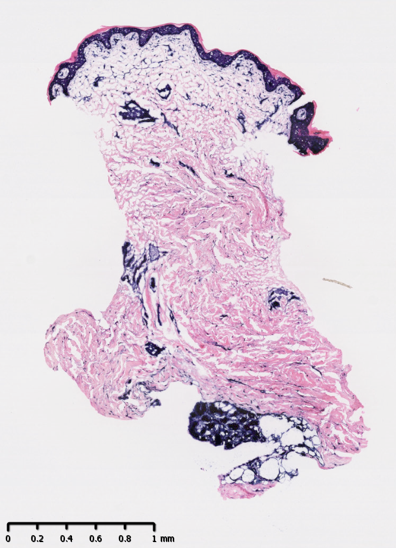

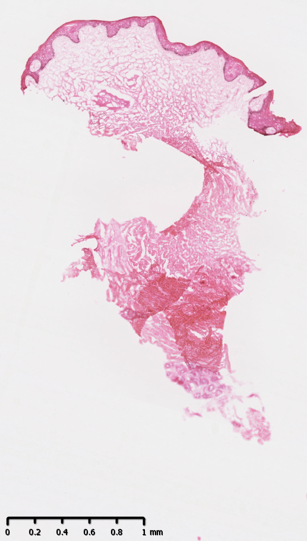


(a)

(b)

(d)

(c)


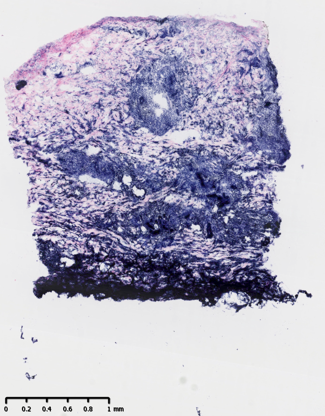

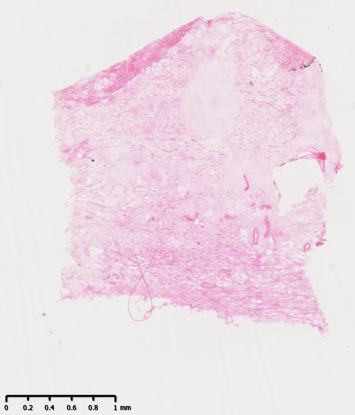

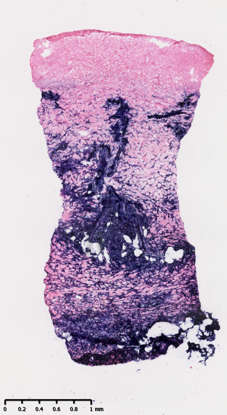

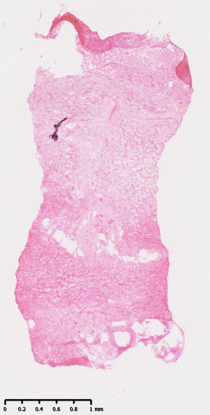


(h)

(e)

(f)

(g)

**Figure 2:** Non-burn skin. Full LDH staining protocol (a), without substrate (lactic acid) (b). Full protocol (c), without nicotine adenine dinucleotide (NAD) (d).

Burn skin. Full LDH staining protocol (e), without substrate (f), Full protocol (g), without NAD (h).

****Supporting information 3: Figure 3.**

(g)

(c)

(a)

(h)

(d)

(i)

(e)

(b)

(j)

(f)

**Supporting information 3: Table 1.**

| Name | Total Area (pixels) | Percentage of LDH across whole tissue |
| --- | --- | --- |
| Burn Background Whole | 661271 |  |
| Burn Background Superficial | 231023 |  |
| Burn Background Middle | 239389 |  |
| Burn Background Deep | 192057 |  |
| Burn LDH Whole | 247051 | 37.36 |
| Burn LDH Superficial | 87567 | 37.90 |
| Burn LDH Middle | 89621 | 37.44 |
| Burn LDH Deep | 69854 | 36.37 |
| Control Background Whole | 402816 |  |
| Control Background Superficial | 171232 |  |
| Control Background Middle | 159493 |  |
| Control Background Deep | 72574 |  |
| Control LDH Whole | 22582 | 5.61 |
| Control LDH Superficial | 20701 | 12.09 |
| Control LDH Middle | 1775 | 1.11 |
| Control LDH Deep | 113 | 0.16 |

Image analysis using ImageJ was performed and an example of images generated is shown in **Figure** **3**. Original image (a – burn, b - control) of LDH stain. Colour deconvolution using macro to separate out background eosin stain (c – burn, e – control) from LDH stain (d – burn, f – control). Homogenisation of these images (g to j) which undergo pixel count. Note 4.4 pixels = 1µm^2^. Faint blue box indicates delineation of superficial, middle and deep thirds of tissue used for further calculations.

**Table 1** demonstrating example of pixel count and percentage calculation example from patient as in supporting information 3 figure 3**.** Percentage of tissue occupied by LDH positive staining calculated by total pixels LDH staining divided by total pixels background stain multiplied by 100.

**Supporting information 4: Table 2**

| **Tissue Type** | **Region** | **Slope** | **R^2^** | **P-value** |
| --- | --- | --- | --- | --- |
| Burn | Whole | 1.85 | 0.29 | *<0.001 |
| Burn | Superficial third | 1.01 | 0.08 | 0.08 |
| Burn | Middle third | 2.66 | 0.43 | *<0.001 |
| Burn | Deep third | 1.78 | 0.18 | *0.007 |
| Control | Whole | -0.269 | 0.02 | 0.36 |
| Control | Superficial third | -0.18 | 0.01 | 0.57 |
| Control | Middle third | -0.317 | 0.03 | 0.27 |
| Control | Deep third | -0.517 | 0.04 | 0.22 |

**Table 2:** Table providing supporting values for Figure 2B in main manuscript.

**Supporting information 4: Table 3**

| Region | Cause | Median | IQR | p-value (Kruskal-Wallis test) |
| --- | --- | --- | --- | --- |
| Whole tissue | Contact (N=8) | 30.4 | 22.6 – 39.6 | 0.081 |
|  | Flame (N=9) | 52.9 | 49.8 – 59.2 |  |
|  | Scald (N=21) | 24.3 | 17.5 – 35.3 |  |
| Superficial third | Contact (N=8) | 19.3 | 15.6 – 30.7 | 0.151 |
|  | Flame (N=9) | 41.2 | 16.3 – 44.9 |  |
|  | Scald (N=21) | 13.7 | 4.3 – 31.4 |  |
| Middle third | Contact (N=8) | 32.9 | 26.3 – 42.2 | 0.136 |
|  | Flame (N=9) | 54.7 | 36.7 – 69.3 |  |
|  | Scald (N=21) | 28.8 | 16.7 – 44.2 |  |
| Deep third | Contact (N=8) | 41.1 | 28.3 – 53.3 | 0.161 |
|  | Flame (N=9) | 54.5 | 46.2 – 58.9 |  |
|  | Scald (N=21) | 33.4 | 11.9 – 49.5 |  |

**Table 3:** Relationship between cause of Burn and average LDH % across whole tissue, superficial, middle and deep third of tissue. Kruskal wallis test performed to obtain p-value.

**Supporting information 5: Table 4. Control sample LDH stain percentage across tissue**

| **Non-burn Patient number** | ***Age (Yrs)*** | ***Gender*** | ***Type of Burn*** | ***Age of Burn (days)*** | Whole tissue LDH % | sup 3rd LDH % | mid 3rd LDH % | deep 3rd LDH % |
| --- | --- | --- | --- | --- | --- | --- | --- | --- |
| **16C** | 41 | M | Scald | 1 | 11.8 | 18.14 | 13.83 | 2.38 |
| **28C** | 31 | M | Scald | 2 | 8.25 | 19.36 | 1.01 | 2.53 |
| **39C** | 33 | M | Scald | 2 | 15.28 | 19.18 | 14.62 | 11.2 |
| **36C** | 58 | M | Flame | 2 | 15.18 | 28.68 | 5.19 | 9.5 |
| **1C** | 26 | M | Contact | 3 | 36.21 | 39.85 | 32.49 | 35.99 |
| **40C** | 25 | F | Flame | 3 | 5.63 | 14.01 | 1.45 | 0 |
| **35C** | 87 | M | Scald | 3 | 9.55 | 16.46 | 2.73 | 9.48 |
| **34C** | 33 | M | Scald | 4 | 17.29 | 16.91 | 10.37 | 28.77 |
| **22C** | 19 | F | Scald | 4 | 44.84 | 52.64 | 26.17 | 58.36 |
| **30C** | 42 | M | Scald | 4 | 9.41 | 19.98 | 1.62 | 2.12 |
| **7C** | 23 | F | Scald | 4 | 15.88 | 34.58 | 15.34 | 2.75 |
| **15C** | 85 | F | Contact | 4 | 25.79 | 31.11 | 22.97 | 22.51 |
| **25C** | 58 | F | Contact | 5 | 14.82 | 27.66 | 9.23 | 4.81 |
| **4C** | 67 | M | Scald | 5 | 15.61 | 32.22 | 14.66 | 10.52 |
| **13C** | 23 | F | Contact | 5 | 38.49 | 47.39 | 23.42 | 51.13 |
| **18C** | 31 | M | Contact | 5 | 10.64 | 26.91 | 2.29 | 2 |
| **32C** | 58 | F | Scald | 5 | 40.62 | 45.41 | 35.28 | 41.64 |
| **6C** | 23 | M | Contact | 7 | 10.55 | 30.7 | 5.98 | 3.17 |
| **27C** | 55 | M | Scald | 7 | 12.72 | 30.95 | 5.47 | 3.83 |
| **38C** | 58 | F | Scald | 7 | 26.89 | 28.96 | 34.6 | 5.92 |
| **37C** | 53 | M | Scald | 7 | 10.29 | 14.36 | 4.31 | 5.96 |
| **29C** | 71 | M | Scald | 8 | 10.31 | 16 | 3.54 | 4.5 |
| **20C** | 60 | M | Flame | 9 | 17.49 | 32.32 | 9.72 | 4.74 |
| **21C** | 61 | F | Scald | 9 | 9.06 | 13.84 | 7.91 | 1.42 |
| **31C** | 28 | M | Flame | 9 | 20.01 | 29.94 | 14.31 | 18.39 |
| **5C** | 31 | M | Scald | 9 | 23.68 | 40.38 | 13.98 | 9.12 |
| **14C** | 50 | F | Scald | 10 | 23.4 | 33.57 | 27.38 | 0.2 |
| **19C** | 55 | M | Flame | 11 | 5.6 | 12.08 | 1.11 | 0.15 |
| **9C** | 63 | M | Contact | 11 | 5.8 | 11.72 | 4.96 | 2.17 |
| **41C** | 26 | M | Scald | 13 | 10.15 | 18.39 | 2.3 | 6.19 |
| **33C** | 41 | F | Flame | 13 | 13.43 | 21.3 | 4.22 | 14.72 |
| **24C** | 45 | F | Contact | 14 | 25.51 | 34.24 | 18.42 | 24.54 |
| **12C** | 24 | F | Scald | 15 | 10.27 | 21.29 | 5.93 | 1.67 |
| **11C** | 76 | M | Scald | 15 | 18.14 | 24.38 | 15.97 | 3.46 |
| **17C** | 84 | M | Flame | 17 | 14.02 | 27.48 | 7.77 | 1.98 |
| **42C** | 37 | M | Scald | 17 | 8.39 | 14.62 | 6.17 | 1.96 |
| **26C** | 70 | M | Flame | 19 | 27.63 | 46.83 | 15.97 | 13.66 |
| **8C** | 24 | F | Flame | 26 | 9.61 | 18.21 | 2.46 | 10.16 |

**Table 4:** LDH stain percentage across tissue compared against eosin staining for whole tissue, superficial third (sup 3^rd^), middle third (mid 3^rd^) and deep third (deep 3^rd^) for uninjured control skin. Patient number, age of patient, gender, type of injury, age of burn (in days).

**Supporting information 5: Table 5. Burn sample LDH stain percentage across tissue**

| **Burn Patient number** | ***Age (Yrs)*** | ***Gender*** | ***Type of Burn*** | ***Age of Burn (days)*** | **Whole tissue LDH %** | **sup 3rd LDH %** | **mid 3rd LDH %** | **deep 3rd LDH %** |
| --- | --- | --- | --- | --- | --- | --- | --- | --- |
| **16B** | 41 | M | Scald | 1 | 18.14 | 31.37 | 7.34 | 21.15 |
| **28B** | 31 | M | Scald | 2 | 2.66 | 0.73 | 2.95 | 3.6 |
| **39B** | 33 | M | Scald | 2 | 19.22 | 14.07 | 18.54 | 32.29 |
| **36B** | 58 | M | Flame | 2 | 11.3 | 4.93 | 20.04 | 6.6 |
| **1B** | 26 | M | Contact | 3 | 17.18 | 8.15 | 28.37 | 7.19 |
| **40B** | 25 | F | Flame | 3 | 17.45 | 10.76 | 14.7 | 30.12 |
| **35B** | 87 | M | Scald | 3 | 10.8 | 4.2 | 9.25 | 24.5 |
| **34B** | 33 | M | Scald | 4 | 18.62 | 14.32 | 18.31 | 34.97 |
| **22B** | 19 | F | Scald | 4 | 35.1 | 53.03 | 20.15 | 3.6 |
| **30B** | 42 | M | Scald | 4 | 26.63 | 13.69 | 33.24 | 40.31 |
| **7B** | 23 | F | Scald | 4 | 21.48 | 2.94 | 23.86 | 39.95 |
| **15B** | 85 | F | Contact | 4 | 35.33 | 26.68 | 28.8 | 59.4 |
| **25B** | 58 | F | Contact | 5 | 26.55 | 18.31 | 24.68 | 47.16 |
| **4B** | 67 | M | Scald | 5 | 41.86 | 16.82 | 46.19 | 61.16 |
| **13B** | 23 | F | Contact | 5 | 71.6 | 91.28 | 55.22 | 65.12 |
| **18B** | 31 | M | Contact | 5 | 33.26 | 29.84 | 27.99 | 45.75 |
| **32B** | 58 | F | Scald | 5 | 61.03 | 34.23 | 74.58 | 81.94 |
| **6B** | 23 | M | Contact | 7 | 27.48 | 20.32 | 38.34 | 21.62 |
| **27B** | 55 | M | Scald | 7 | 15.29 | 5.42 | 7.94 | 33.44 |
| **38B** | 58 | F | Scald | 7 | 49.66 | 41.13 | 56.61 | 49.52 |
| **37B** | 53 | M | Scald | 7 | 28.06 | 26.45 | 28.58 | 30.5 |
| **29B** | 71 | M | Scald | 8 | 24.3 | 7.3 | 41.39 | 31.98 |
| **20B** | 60 | M | Flame | 9 | 49.83 | 44.89 | 48.38 | 57.67 |
| **21B** | 61 | F | Scald | 9 | 31.94 | 27.6 | 48.97 | 11.87 |
| **31B** | 28 | M | Flame | 9 | 22.96 | 4.07 | 32.01 | 34.58 |
| **5B** | 31 | M | Scald | 9 | 51.56 | 64.53 | 36.73 | 46.19 |
| **14B** | 50 | F | Scald | 10 | 1.52 | 0 | 0.65 | 4.45 |
| **19B** | 55 | M | Flame | 11 | 37.36 | 37.9 | 37.43 | 36.37 |
| **9B** | 63 | M | Contact | 11 | 52.87 | 16.28 | 69.26 | 76.66 |
| **41B** | 26 | M | Scald | 13 | 53.78 | 25.05 | 54.72 | 78.71 |
| **33B** | 41 | F | Flame | 13 | 10.4 | 4.34 | 16.69 | 7.09 |
| **24B** | 45 | F | Contact | 14 | 49.34 | 31.6 | 58.73 | 59.4 |
| **12B** | 24 | F | Scald | 15 | 44.23 | 23.2 | 44.19 | 66.77 |
| **11B** | 76 | M | Scald | 15 | 23.95 | 3.24 | 36.06 | 45.26 |
| **17B** | 84 | M | Flame | 17 | 62.44 | 41.2 | 88.12 | 54.5 |
| **42B** | 37 | M | Scald | 17 | 71.12 | 44.17 | 81.75 | 88.82 |
| **26B** | 70 | M | Flame | 19 | 59.59 | 55.22 | 68.06 | 50.48 |
| **8B** | 24 | F | Flame | 26 | 59.23 | 44.84 | 74.62 | 58.91 |

**Table 5:** LDH stain percentage across tissue compared against eosin staining for whole tissue, superficial third (sup 3^rd^), middle third (mid 3^rd^) and deep third (deep 3^rd^) for burn skin. Patient number, age of patient, gender, type of injury, age of burn (in days).

**Supporting information 6: Macro for image analysis of LDH and eosin stained tissue:**

The following code was created for image analysis and applied to all raw images to obtain LDH percentage across the regions of tissue. Macro created in ImageJ. Prior to running code, calibration of length in raw images was performed at 4 pixels per μm.

**Code starts:**

*roiManager("reset");*

*run("Select None");*

*waitForUser(""," Select original image \n \n then press ok");*

*//waitForUser(""," Select whole tissue \n \n then press ok");*

*fn=getTitle();*

*run("Duplicate...", "title=wt");*

*run("8-bit");*

*rename(fn+" whole tissue");*

*run("Smooth");*

*run("Threshold...");*

*setAutoThreshold("Triangle");*

*waitForUser("","Check threshold for whole tissue");*

*run("Analyze Particles...", "size=50-Infinity summarize");*

*waitForUser(""," Draw rectangle \n \n then press ok");*

*getSelectionBounds(x, y, width, height);*

*makeRectangle(x, y, width, height/3);*

*roiManager("add");*

*roiManager("select", 0);*

*roiManager("rename", "Sup");*

*rename(fn+" whole tissue Sup");*

*run("Analyze Particles...", "size=50-Infinity summarize");*

*makeRectangle(x, y+height/3, width, height/3);*

*roiManager("add");*

*roiManager("select", 1);*

*roiManager("rename", "Mid");*

*rename(fn+" whole tissue Mid");*

*run("Analyze Particles...", "size=50-Infinity summarize");*

*makeRectangle(x, y+(2*(height/3)), width, height/3);*

*roiManager("add");*

*roiManager("select", 2);*

*roiManager("rename", "Deep");*

*rename(fn+" whole tissue Deep");*

*run("Analyze Particles...", "size=50-Infinity summarize");*

*waitForUser(""," Select LDH tissue \n \n then press ok");*

*rename(fn+" LDH stain tissue");*

*setAutoThreshold("Default");*

*waitForUser("","Check threshold for LDH");*

*run("Analyze Particles...", "size=50-Infinity summarize");*

*roiManager("select", 0);*

*rename(fn+" LDH stain Sup");*

*run("Analyze Particles...", "size=50-Infinity summarize");*

*roiManager("select", 1);*

*rename(fn+" LDH stain Mid");*

*run("Analyze Particles...", "size=50-Infinity summarize");*

*roiManager("select", 2);*

*rename(fn+" LDH stain Deep");*

*run("Analyze Particles...", "size=50-Infinity summarize");*

*waitForUser(""," Select original image \n \n then press ok");*

*fn2=getTitle();*

*roiManager("Show All");*

*path=File.directory;*

*//save drawing or image*

*saveAs("Tiff",path+fn2 + " overlay");*

**Code ends.**
